# Supplementary material for: High frequency of potential phosphodiesterase type 5 inhibitor drug interactions in males with HIV infection and erectile dysfunction
Source: PLoS One. 2021 May 6;16(5):e0250607. doi: 10.1371/journal.pone.0250607 (PMC8101910; doi:10.1371/journal.pone.0250607)
Supplement: Attachment — Submitted filename: Response to Reviewers PDE5-ART DDI (2021-03-28).doc [file pone.0250607.s001.doc]

**Response to Academic Editor and Review Comments – PONE-D-20-32474**

***“High Frequency of Potential Phosphodiesterase Type 5 Inhibitor Drug Interactions in Males with HIV Infection and Erectile Dysfunction”***

**Editor:**

| **Comments** | | **Response** | **Manuscript Change** |
| --- | --- | --- | --- |
| 1 | Cases and controls were selected from the cohort. Cases were those who had DDIs as defined. The controls were those without the DDIs. Given that several factors may influence DDIs it is important for the authors to elaborately describe the cases and whether the controls were matched or unmatched?  Were there other co-morbid conditions and medications apart from ART? Were these matched? Case control design is used when a condition is rare/uncommon, however in this manuscript, the cases were actually more than the controls in >3:1. | We have clarified throughout the manuscript that the logistic regression analysis was performed on two separate cohorts consisting of patients who met the DDI definition versus those who did not (non-DDI cohort). We have deleted the words “cases” and “controls” throughout. We agree that use of such terms implies a case-control study design rather than a cohort design.  We agree that the high number of patients who met the DDI definition was unexpected and believe that a critical reason to publish these data is to increase awareness of the well-described clinically relevant drug interaction between PDE-5 inhibitors and specific ART. | Page 2, Lines 34-35 now read, “A secondary logistic regression analysis was performed on those with and without DDIs to identify factors associated with initial DDI episode.”  Page 6, Lines 119-121: “In order to determine what factors were associated with the presence of a DDI, patients were divided into a DDI and a non-DDI cohort for logistic regression analysis. |
| 3 | Patient characteristics Page 5 Line 75 mentions co-morbidities however no information on the co-morbid conditions and the associated medications is provided in the results section.  PDE 5 inhibitors is known to have interactions with not only ART but antibiotics like erythromycin, clarithromycin, antifungals, hypertensive medications, alcohol etc. There is no evidence presented on these associated factors in the manuscript. | We agree that this information is important and have included comorbid condition information in the manuscript.  We agree that more comprehensive medication prescription information should be included and have described two instances in the DDI cohort where co-prescribed medications may have further increased PDE-5 inhibitor serum concentrations via 3A4 inhibition. This information further underpins the importance of this manuscript as it highlights a drug interaction that appears to be unrecognized in clinical settings where men with HIV and ED are receiving PDE-5 inhibitors.  We have also noted that there were no patients identified in the DDI cohort who were co-prescribed medications that may lower PDE-5 serum concentrations such as rifampin, phenytoin, or carbamazepine. | Page 5, Lines 93-95: “While all patients were diagnosed with ED, collected comorbidities only included those previously identified as posing a high risk for severe ED such as cardiovascular disease, mental health disorders, diabetes, and smoking history.  Table 1 now includes comorbidities.  Pages 5-6, Lines 97-100: “Additional medications known to affect CYP3A4 metabolism were captured if co-prescribed within 30-days of PDE-5 inhibitor dispensing date. These included 3A4 inhibitors (macrolides, azole antifungals, and statins) or those with 3A4 induction potential (rifamycins and anti-epileptic drugs).”  Page 8, Lines 149-157: “In two patients in the DDI cohort, simvastatin prescriptions were dispensed along with sildenafil and RTV-boosted regimens. As a CYP3A4 inhibitor, simvastatin would be expected to further exacerbate the PDE-5 inhibitor-RTV interaction. In both instances, the simvastatin prescription was discontinued and replaced by an alternative statin without CYP3A4 interaction potential. In one of these patients, one additional 90-day supply sildenafil prescriptions were dispensed. In the second patient, sildenafil was switched to vardenafil at an inappropriate dose of 10 mg. No patients in the non-DDI group received additional prescriptions known to affect CYP3A4 metabolism within 30 days of the co-dispensing of a PDE-5 inhibitor and potentially-interacting ART.” |
| 4 | Definition of DDI: The authors define a DDI was documented if: 1) the initial sildenafil dose exceeded 25 mg, 2) the initial vardenafil or tadalafil dose exceeded 2.5 mg, or 3) the tadalafil dose exceeded 10 mg at any time during the co-administration period. For those receiving a PDE-5 inhibitor who received a new first prescription for a potentially-interacting ART regimen, a DDI was identified if the PDE-5 inhibitor dose was not discontinued or the dose was not reduced within 30 days of the new prescription for any PI-based ART (RTV-boosted or un-boosted) or COBI-boosted ART. The scientific basis of this criteria is unclear. For example Sildenafil is given at a dose from 25mg to 100mg to get the desirable effect. This may be due to specific patient related factors or idiosyncratic. It is therefore problematic to assume that a dose of more than 25mg may be a potential case of DDI. The criteria of the dose not discontinued or reduced should be adequately explained.  Review of point 1 to 4 may influence the outcomes/results and subsequently the conclusion | We clarified the DDI definition to specify that these PDE-5 dosing criteria were only applied when co-dispensed with ART known to increase PDE-5 serum concentrations via CYP450 3A4 inhibition (e.g. ritonavir, cobicistat, etc.). We took these criteria from two clinical practice guidelines and two drug interaction drug information references and cited these references in the manuscript.  In the background, we added the well-known mechanism of the drug interaction and added referenced statements that clearly delineate how co-administration of strong CYP450 3A4 inhibitors significantly increase PDE-5 inhibitor exposure, which are CYP450 3A4 substrates.  We agree that dose titration to desired effect should be patient-specific. However, multiple clinical practice guidelines, drug interaction analysis software programs, and drug information resources give specific and consistent recommendations on starting doses of PDE-5 inhibitors when co-administered with potentially-interacting ART. These details remain in the manuscript.  This is also the reason why different definitions were applied to patients that received an initial PDE-5 inhibitor prescription on previous ART versus those who had previously been receiving dose-titrated up on PDE-5 inhibitors that received new prescriptions for potentially-interacting ART. We have emphasized that doses were 2- or 4-fold higher than recommended by multiple clinical practice guidelines. | Page 4, Lines 58-71: “Given that each of these PDE-5 inhibitors is primarily metabolized by the cytochrome P450 3A4 isoenzyme (CYP3A4), drug information databases and antiretroviral therapy (ART) guidelines warn against co-administration with strong CYP3A4 inhibitors [4-7]. Protease inhibitors (PIs) and pharmacokinetic boosters such as ritonavir (RTV) and cobicistat (COBI) strongly inhibit CYP3A4 drug metabolism [6,8,9]. Therefore, resources recommend PDE-5 inhibitor dose reduction when initiating therapy with these potentially-interacting ART regimens or recommend lower starting PDE-5 inhibitor doses in patients already receiving this ART to avoid excessive PDE-5 inhibitor exposure. These recommendations are not only based on the theoretical mechanism of this CYP3A4-mediated interaction, but also on pharmacokinetic studies confirming substantially-elevated PDE-5 inhibitor levels with co-administration of PIs, RTV, or COBI. A four-fold increase in sildenafil systemic exposure results when given with PIs alone or RTV-boosted PIs. A greater than two-fold increase in sildenafil maximum serum concentrations was observed with COBI-boosted ART co-administration [10-12].  Page 6, Lines 104-106: “A DDI was identified when a PDE-5 inhibitor was co-dispensed within 30 days of potentially-interacting ART using the following consistent definitions found across published guidelines and drug interaction programs [4-7].  Page 15, Lines 187-190: “At least two published case reports describe deaths associated with DDIs involving PDE-5 inhibitors and RTV-boosted ART [23,25]. Such consequences make the recommendations for PDE-5 inhibitor dose reduction and slow up-titration essential when these ED agents are co-prescribed with strong CYP 3A4 inhibitors such as PIs, RTV, and COBI.  Page 15, Lines 194-198: “In addition, approximately 60% of these patients received a PDE-5 inhibitor dose that was more than 4 to 8 times higher than the recommended starting dose. This would yield initial systemic exposures greater than 10 times higher than maximum recommended PDE-5 inhibitor doses when given alone.” |
| 5 | Additional DDI captured for gaps in medication should adequately elaborated Page 5 Line 89 | We agree and have added additional information to adequately describe how these data were collected. | Page 6, Lines 114-117: “The duration of a single discrete DDI episode was calculated by using the days supply listed on the prescription record. An additional DDI episode was captured if the aforementioned DDI definitions were met following a gap in prescription overlap greater than 90 days following the documented days supply.” |
| 6 | Description of the metabolic pathway/mechanism of action: The introduction must include why RTV or COBI is likely to increase the levels of PDE 5 inhibitors. Is it through the cytochrome P450 enzyme complex or another mechanism? This should be described. | We agree and have added additional information describing the mechanism of the drug interaction and confirmatory pharmacokinetic studies in the background section. | Page 4, Lines 58-63: “Given that each of these PDE-5 inhibitors is primarily metabolized by the cytochrome P450 3A4 isoenzyme (CYP3A4), drug information databases and antiretroviral therapy (ART) guidelines warn against co-administration with strong CYP3A4 inhibitors [4-7]. Protease inhibitors (PIs) and pharmacokinetic boosters such as ritonavir (RTV) and cobicistat (COBI) strongly inhibit CYP3A4 drug metabolism [6,8,9].” |
| 8 | Please ensure that your manuscript meets PLOS ONE's style requirements, including those for file naming | We have ensured that the manuscript meets PLOS ONE’s style requirements and file naming conventions. |  |
| 9 | Please provide a justification as to why participant ethnicity was studied as a variable. | We agree and have including a justification. | Page 5, Lines 91-93: “Race and ethnicity data were collected because previous studies have identified differences in the risk factors for and prevalence of severe erectile dysfunction [21].” |
| 10 | Furthermore, please note that according to our submission guidelines. Outmoded terms and potentially stigmatizing labels should be changed to more current, acceptable terminology. For example: “Caucasian” should be changed to “white” or “of [Western] European descent” (as appropriate). | We agree and have changed all terminology to more current acceptable terms. |  |
| 11 | Finally please provide a citation to the guidelines which were used to identify DDIs [line 81]. | We have added 4 citations used to identify DDIs. | Page 6, Lines 104-106: “A DDI was identified when a PDE-5 inhibitor was co-dispensed within 30 days of potentially-interacting ART using the following consistent definitions found across published guidelines and drug interaction programs [4-7].” |

**Reviewer:**

| **Comments** | | **Response** | **Manuscript Change** |
| --- | --- | --- | --- |
| 1 | This study reported on drug-drug interaction (DDI) between phosphodiesterase type 5 (PDE-5) inhibitor and antiretroviral therapy (ART) in males with HIV infection and erectile dysfunction (ED) diagnosis. Generally, in this retrospective nature of the present study, causality cannot be inferred. | We agree that causality cannot be inferred and have used the word “association” or “associated with” throughout the manuscript. |  |
| 2 | They selected patients receiving both treatments among patients with HIV infection and ED, and defined those with high doses of PDE-5 inhibitor by a DDI group. Such patients may require its high doses due to disease severity or patient potential confounders, and I wonder why they have a conclusion that these patients met ED due to DDIs or they assessed other DDIs-related adverse effects such as hypotension and syncope. | We agree that high ED severity may warrant high PDE-5 doses. However, multiple clinical practice recommend lower initial starting doses since the CYP3A4-mediated inhibition of PDE-5 inhibitors will result in a greater than 2- to 4-fold increase in systemic PDE-5 inhibitor exposure.  We have clarified that we did not assign a diagnosis of ED based on DDIs, PDE-5 inhibitor use, or PDE-5 inhibitor dose. All patients in this study had a documented diagnosis of ED without regard for PDE-5 inhibitor use. | Page 4, Lines 63-66: “Therefore, resources recommend PDE-5 inhibitor dose reduction when initiating therapy with these potentially-interacting ART regimens or recommend lower starting PDE-5 inhibitor doses in patients already receiving this ART to avoid excessive PDE-5 inhibitor exposure.”  Page 15, Lines 188-190: “Such consequences make the recommendations for PDE-5 inhibitor dose reduction and slow up-titration essential when these ED agents are co-prescribed with strong CYP 3A4 inhibitors such as PIs, RTV, and COBI.” |
| 3 | Additionally, how many patients taking both PDE-5 inhibitor and ART did not meet ED during the same period? Among these patients without ED, how many patients were considered as DDIs group, namely high doses of PDE-5 inhibitor?  More detailed description regarding interaction between these treatments and adverse effects should be addressed. Otherwise, authors should downplay the interpretation of their results. | We have clarified that all patients in this study had a documented diagnosis of ED and were taking both ART and a PDE-5 inhibitor.  We agree and have added additional information describing the mechanism of the drug interaction and confirmatory pharmacokinetic studies in the background section. | Page 5, Lines 86-89: “The NHS database was queried to identify male participants with a diagnosis of ED recorded between 2001 and 2016. Those with an ED diagnosis receiving a prescription for a PDE-5 inhibitor (sildenafil, vardenafil, or tadalafil) and a potentially-interacting ART regimen co-dispensed within 30 days were included.”  Page 5, Lines 93-95: “While all patients were diagnosed with ED, collected comorbidities only included those previously identified as posing a high risk for severe ED such as cardiovascular disease, mental health disorders, diabetes, and smoking history.”  Page 4, Lines 58-71: “Given that each of these PDE-5 inhibitors is primarily metabolized by the cytochrome P450 3A4 isoenzyme (CYP3A4), drug information databases and antiretroviral therapy (ART) guidelines warn against co-administration with strong CYP3A4 inhibitors [4-7]. Protease inhibitors (PIs) and pharmacokinetic boosters such as ritonavir (RTV) and cobicistat (COBI) strongly inhibit CYP3A4 drug metabolism [6,8,9]. Therefore, resources recommend PDE-5 inhibitor dose reduction when initiating therapy with these potentially-interacting ART regimens or recommend lower starting PDE-5 inhibitor doses in patients already receiving this ART to avoid excessive PDE-5 inhibitor exposure. These recommendations are not only based on the theoretical mechanism of this CYP3A4-mediated interaction, but also on pharmacokinetic studies confirming substantially-elevated PDE-5 inhibitor levels with co-administration of PIs, RTV, or COBI. A four-fold increase in sildenafil systemic exposure results when given with PIs alone or RTV-boosted PIs. A greater than two-fold increase in sildenafil maximum serum concentrations was observed with COBI-boosted ART co-administration [10-12]. |
| 5 | Patients needed higher doses of these disease modifying treatments might be likely to have sicker HIV conditions; hence this drug-drug interaction could be indispensable for managing such HIV patients. However, their description table lacked patient factors. | We agree and have added patient co-morbidities to our table and logistic regression models. We would also highlight our inclusion of HIV duration and ED duration as a factors associated with DDIs. | Page 5, Lines 93-95: “While all patients were diagnosed with ED, collected comorbidities only included those previously identified as posing a high risk for severe ED such as cardiovascular disease, mental health disorders, diabetes, and smoking history.”  Page 6-7, Lines 119-127: “In order to determine what factors were associated with the presence of a DDI, patients were divided into a DDI and a non-DDI cohort for logistic regression analysis. The following factors for a first DDI occurrence were evaluated using univariate logistic regression: age, race/ethnicity, time from HIV diagnosis to first DDI, time from ED diagnosis to first DDI, comorbidities that increase risk for more severe ED, first PDE-5 inhibitor prescription after RTV- or COBI-boosted ART, time from HIV diagnosis to first DDI, and time from ED diagnosis to first DDI. Factors with a p-value less than 0.2 in univariate analysis were included in a multivariate logistic regression model to identify associations associated with first DDI occurrence.”  These comorbidities and additional patient factors have been included in both Tables 1 and 2. |
| 6 | In multiple logistic regression model, all retained variables were medication factors (RTV-boosted atazanavir, Sildenafil, RTV-boosted, darunavir, RTV-boosted PI or PI-based ART). I think that these variables may be strongly correlated since patients with more disease severity require higher doses of these HIV-related treatments. Did you look at the multicollinearity of such medication variables? | We agree and thank the reviewer for this insight. We have included co-morbidities in the analysis and retained age, HIV duration, and ED duration in the models. After re-analyzing, three patient factors were retained. | Page 13, Lines 169-171: “Among participant factors in univariate analyses that met criteria for inclusion in the multivariate model, age less than 45 and major depressive disorder were associated with increased odds of DDIs; whereas dyslipidemia was associated with decreased DDI risk (Table 2).” |
